# Revision outcomes after failed hip resurfacing arthroplasty vary by failure indication and component strategy: A systematic review

**DOI:** 10.1002/jeo2.70965

**Published:** 2026-11-03

**Authors:** Srikar Reddy Namireddy, Hariharan Subbiah Ponniah, Milos Brjklac, Justin Cobb, Kartik Logishetty

**Affiliations:** ^1^ MSk Lab, Imperial College London London UK; ^2^ Cleveland Clinic London Hospital London UK; ^3^ Young Adult Hip Unit University College London Hospitals NHS Foundation Trust London UK; ^4^ Division of Surgery and Interventional Science University College London London UK

**Keywords:** ARMD, ceramic‐on‐ceramic, hip resurfacing arthroplasty (HRA), HRA revision, metal‐on‐metal

## Abstract

**Purpose:**

Hip resurfacing arthroplasty (HRA) is a bone‐conserving procedure indicated for end‐stage osteoarthritis in younger, active patients. The widespread failure of metal‐on‐metal (MoM) bearings due to adverse reactions to metal debris has generated a large and complex revision cohort. The recent regulatory approval of ceramic‐on‐ceramic (CoC) resurfacing devices necessitates an updated synthesis of HRA revision outcomes.

**Methods:**

Following Preferred Reporting Items for Systematic Reviews and Meta‐Analyses guidelines, a systematic search of MEDLINE, EMBASE, CINAHL, Cochrane Library and HMIC was conducted. Studies reporting outcomes of primary HRA revision with a minimum of 12 months of follow‐up and extractable re‐revision or functional outcome data were included. Studies reporting primary HRA survivorship only were excluded. Data were extracted on revision strategy, indications, serum metal ion levels, patient‐reported outcome measures and re‐revision rates.

**Results:**

Twenty‐three studies were included, encompassing 2320 reported HRA revision procedures. Across 1705 hips with longitudinal data, 157 re‐revisions or major reoperations occurred (9.2%). Both‐component revision had the lowest crude strategy‐specific rate (59/828; 7.1%), followed by femoral‐only revision (63/486; 13.0%) and isolated acetabular revision (10/52; 19.2%). Strategy was indication‐dependent: femoral‐only revision predominated for fracture (239/325; 73.5%), whereas both‐component revision predominated for metal‐related pathology (245/368; 66.6%) and loosening/osteolysis (268/416; 64.4%). Post‐revision, cobalt and chromium concentrations decreased in both studies reporting paired data; HHS improved from 64.4 to 92.5. Instability/dislocation/intraprosthetic dislocation was the commonest complication (32/118; 27.1%), followed by periprosthetic joint infection (24/118; 20.3%). Primary CoC resurfacing had 98.0% 5‐year survivorship, with predominantly mechanical failures and no re‐revision data.

**Conclusions:**

HRA revision can provide functional recovery and metal ion reduction, but outcomes depend on indication, component status and construct. Both‐component revision had the lowest crude observed re‐revision proportion. Femoral‐only revision may be considered when the shell is fixed, well‐oriented, undamaged, and biologically uninvolved. Retained‐cup dual mobility is a component‐preserving option but carries risks of intraprosthetic dislocation and uncertain long‐term polyethylene wear.

**Level of Evidence:**

Level IV.

## Graphical abstract

Revision outcomes following failed hip resurfacing arthroplasty vary according to the underlying failure mechanism and the revision strategy selected. Femoral‐only revision was most commonly used for fracture, whereas both‐component revision predominated in metal‐related pathology and loosening or osteolysis. The observed subsequent‐surgery rates were lowest after both‐component revision, although these findings should be interpreted in the context of indication and component status.

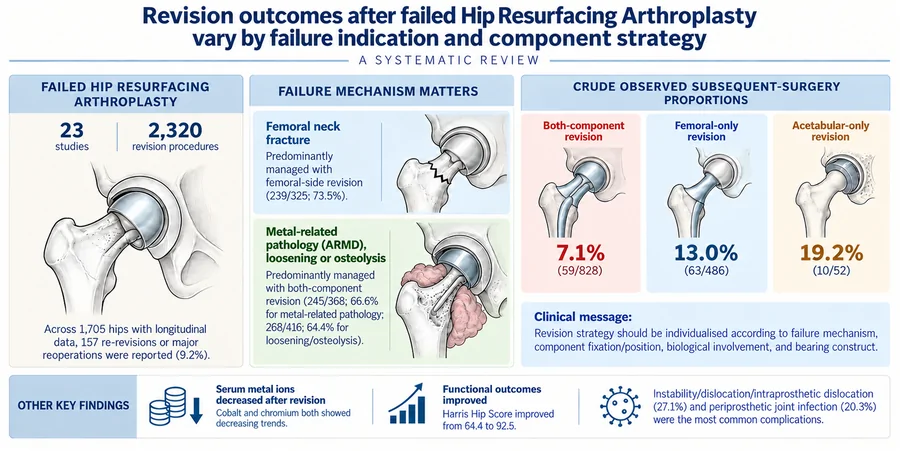

**Abbreviations**
ALTR: adverse local tissue reaction
ALVAL: aseptic lymphocyte‐dominated vasculitis‐associated lesion
AOANJRR: Australian Orthopaedic Association National Joint Replacement Registry
ARMD: adverse reaction to metal debris
BHR: Birmingham hip resurfacing
CoC: ceramic‐on‐ceramic
HHS: Harris Hip Score
HOOS: Hip disability and Osteoarthritis Outcome Score
HRA: hip resurfacing arthroplasty
MCID: minimal clinically important difference
MoM: metal‐on‐metal
NJR: National Joint Registry
OHS: Oxford Hip Score
PJI: periprosthetic joint infection
PRISMA: Preferred Reporting Items for Systematic Reviews and Meta‐Analyses
PROM: patient‐reported outcome measure
THA: total hip arthroplasty
UCLA: University of California, Los Angeles
WOMAC: Western Ontario and McMaster Universities Osteoarthritis Index

## INTRODUCTION

The clinical burden of hip osteoarthritis continues to increase, driven by an ageing population with expectations for sustained physical activity and functional capacity [26]. Total hip arthroplasty (THA) is widely regarded as one of the most successful procedures in modern medicine but may be less suited for younger, very active patients, due to the risk of accelerated bearing wear under high and long‐term cyclical loading, periprosthetic femoral fractures, dislocation and the sacrifice of proximal femoral bone stock [26].

Hip resurfacing arthroplasty (HRA) was developed to address these limitations. Modern HRA was established in the mid‐1990s with the introduction of large‐diameter, highly polished metal‐on‐metal (MoM) articulations, most notably the Birmingham hip resurfacing (BHR) [12, 30]. Compared with THA, HRA offers more physiological load transfer to the proximal femur, preserves femoral bone stock, provides greater inherent stability and may permit return to high‐impact activities [11, 12].

Despite these advantages, wider application of MoM HRA revealed critical vulnerabilities. While registry data confirm survivorship exceeding 94% at 14–17 years in appropriately selected male patients with primary osteoarthritis [4, 12], suboptimal component positioning and the use of smaller‐diameter bearings led to edge‐loading, stripping the lubricating fluid film and driving accelerated cobalt and chromium ion release [13, 25].

The resulting biological response—termed adverse reaction to metal debris (ARMD) or aseptic lymphocyte‐dominated vasculitis‐associated lesion (ALVAL)—involves cell‐mediated and innate immune responses to metal wear products, resulting in local soft‐tissue necrosis, osteolysis, and damage to the abductor musculature [7, 13]. These tissue reactions prompted device recalls (including DePuy ASR, Zimmer Durom), international surveillance protocols, and a marked reduction in HRA utilisation globally. However, failed HRA is not defined by metal‐debris disease alone; femoral neck fracture, component loosening, malposition, infection, instability and persistent pain may each create a different revision problem [22, 41, 45].

The orthopaedic community consequently faced the management of a large cohort requiring revision for failed MoM HRA [41, 45]. Although these procedures are often described collectively as conversion to THA, that term can obscure the operative decision being made [21]. Surgeons must select from a spectrum of strategies—both‐component exchange, isolated femoral revision or isolated acetabular revision—each with distinct biomechanical consequences [18, 21, 22, 45]. In practice, the suitability of each strategy depends on why the resurfacing failed, whether the acetabular shell is fixed and well positioned, and whether the soft‐tissue envelope has been compromised [13, 18, 21, 22, 45].

For isolated femoral revision, a dual mobility polyethylene articulation has emerged as an elegant alternative to a new large‐diameter metal head [6, 15, 31]. Cup retention may preserve acetabular bone stock and reduce operative morbidity, but it also leaves the surgeon dependent on the position, fixation, and bearing compatibility of the retained shell [2, 6, 18]. Conversely, complete component exchange may more definitively address malposition, loosening, or ongoing metal wear, but at the cost of acetabular revision [13, 22, 45].

The landscape is further evolving with regulatory approval of ceramic‐on‐ceramic (CoC) resurfacing—specifically the ReCerf® (MatOrtho) and the H1® (Zimmer Biomet) devices—which offer the biomechanical advantages of resurfacing while eliminating metal‐related toxicological risk [23, 27, 29]. A recent review has summarised survivorship, complications, and functional outcomes after conversion of MoM HRA to THA, but there remains limited guidance on strategy selection if indication for failure and component management are not considered together [21, 38].

The primary aim of this systematic review was to evaluate re‐revision rates, functional outcomes and metal ion normalisation across HRA revision strategies, while examining the tribological trade‐offs between dual mobility and large metal head constructs in isolated femoral revision and contextualising these findings within the emerging evidence for CoC HRA. It was hypothesised that revision outcomes would vary according to failure indication and component management, with component‐preserving strategies most appropriate when the retained component was mechanically robust and biologically uninvolved.

## MATERIALS AND METHODS

### Search strategy and study selection

This systematic review was conducted in accordance with Preferred Reporting Items for Systematic Reviews and Meta‐Analyses (PRISMA) guidelines [33]. The review protocol was prospectively registered on PROSPERO (registration number: CRD420261363918).

A Boolean search strategy was applied across MEDLINE, EMBASE, CINAHL, the Cochrane Central Register of Controlled Trials and HMIC from inception to early 2026. Search terms combined MeSH and free‐text keywords: (‘hip resurfacing’ OR ‘hip resurfacing arthroplasty’ OR ‘surface replacement arthroplasty’ OR ‘metal‐on‐metal hip’ OR ‘ceramic‐on‐ceramic resurfacing’) AND (‘revision’ OR ‘re‐revision’ OR ‘re‐operation’ OR ‘conversion’ OR ‘failure’ OR ‘ARMD’ OR ‘ALVAL’ OR ‘metallosis’). No language or geographic restrictions were applied. Duplicate records were removed using automated and manual deduplication.

### Eligibility criteria

Studies were eligible if they reported clinical, radiographic or toxicological outcomes for adult patients undergoing revision of a primary HRA device, using a PICO framework. The population comprised patients with failed primary HRA; the intervention was any surgical revision strategy; comparators included intra‐cohort strategy comparisons or pre‐ versus post‐operative functional data; and primary outcomes were implant survivorship following revision (re‐revision rate), complication rates, post‐operative functional scores, serum cobalt or chromium concentrations and construct‐specific failure modes. A minimum cohort of five revised hips and at least 12 months of mean post‐revision follow‐up were required.

Studies were excluded if they: (1) aggregated HRA and THA revision data without permitting HRA‐specific data extraction; (2) described surgical technique without reporting longitudinal outcomes; (3) were published only as unverified conference abstracts; or (4) reported solely primary HRA survivorship without separately reporting outcomes of the revision procedures themselves. Studies reporting primary CoC HRA outcomes did not meet eligibility criteria and were not included among the studies forming the systematic revision evidence base. Contemporary primary CoC reports identified during the search were considered separately as contextual literature in the discussion and did not contribute to any revision denominator, event rate or quantitative synthesis.

### Data extraction and quality assessment

Two reviewers independently screened titles and abstracts. Discrepancies were resolved by full‐text review; residual disagreements by consensus with a third reviewer. Data extraction used a standardised proforma and was performed independently by two reviewers. Extracted variables included patient demographics, primary device metallurgy, revision indication, revision strategy, acetabular component management, bearing surfaces used at revision, pre‐ and post‐operative patient‐reported outcome measures (PROMs), longitudinal serum cobalt and chromium concentrations, complication profiles and subsequent re‐revision.

Re‐revision was defined as subsequent revision surgery involving exchange, removal, or addition of a prosthetic component after the index HRA revision. Major reoperation was defined as a subsequent operative procedure after the index revision that did not necessarily involve component exchange, including procedures such as debridement, antibiotics and implant retention (DAIR). Where primary studies did not permit these outcomes to be separated, the reported event was retained as a composite ‘re‐revision or major reoperation’ outcome and was not treated as synonymous with re‐revision.

### Statistical synthesis

Given methodological heterogeneity across included studies—differing outcome metrics, complication classifications, revision indications, component strategies and follow‐up durations—a formal meta‐analysis was not appropriate. Most strategy‐specific reports provided crude arm‐level counts rather than adjusted comparative effects, and pooling simple proportions would not account for censoring or unequal follow‐up. A random‐effects model, irrespective of heterogeneity estimator, would therefore have generated a statistically pooled estimate from clinically and methodologically non‐equivalent observations [36].

Accordingly, quantitative findings were synthesised descriptively. Crude event proportions were calculated only where extractable denominators were available and are explicitly described as crude proportions rather than comparative risks. Follow‐up duration is reported alongside study‐level outcomes wherever available, and Kaplan–Meier estimates, or other time‐to‐event measures are preferentially reported where supplied by the primary study. Registry data from the Australian Orthopaedic Association National Joint Replacement Registry (AOANJRR) and National Joint Registry (NJR) were synthesised narratively as population‐level evidence [4, 8, 41, 45].

Continuous outcomes were not formally pooled when variance estimates or compatible summary statistics were unavailable. Metal‐ion changes are therefore presented by individual study rather than as sample‐size‐weighted pooled values. PROMs were summarised by instrument. Minimal clinically important difference (MCID)‐normalisation was considered [35], but the available revision literature did not provide sufficiently consistent instrument‐specific change estimates and dispersion measures to support valid conversion into pooled MCID units.

## RESULTS

### Study selection and characteristics

The initial database search identified 1124 records. After removal of 388 duplicates, 736 unique abstracts were screened, with 643 excluded. Ninety‐three full‐text articles were assessed for eligibility. Twenty‐three studies met all inclusion criteria, encompassing 2320 reported HRA revision procedures. Seventy were excluded: 64 for inadequate follow‐up, non‐HRA‐specific data or technique‐description only, and 6 studies that reported solely primary HRA survivorship without extractable revision data. The systematic selection process is presented in Figure 1.

**Figure 1:**
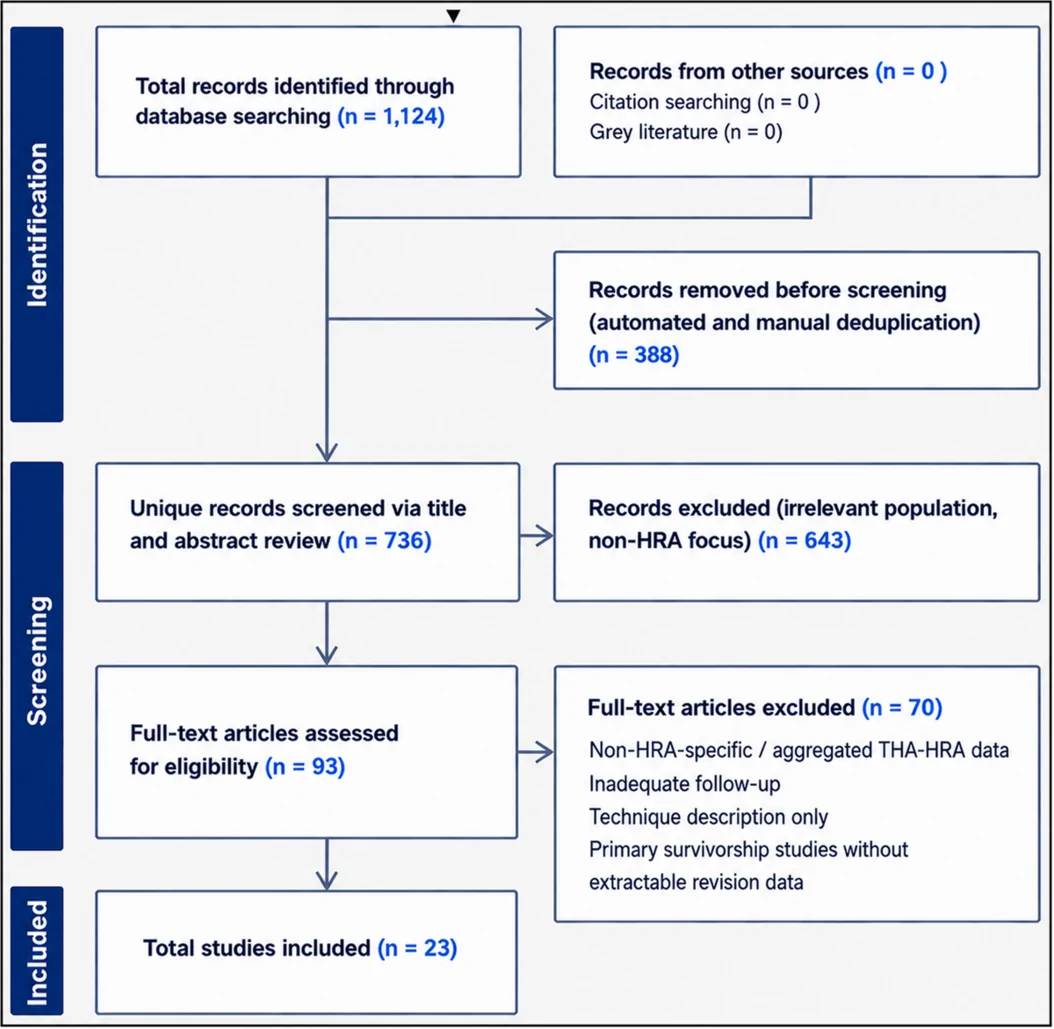
PRISMA (Preferred Reporting Items for Systematic Reviews and Meta‐Analyses) flow diagram. HRA, hip resurfacing arthroplasty; THA, total hip arthroplasty.

The included evidence base comprised 2 national registry analyses, 8 comparative, matched or strategy‐comparative cohort studies and 13 non‐comparative institutional cohort studies or case series. No randomised controlled trials comparing revision strategies after failed HRA were identified. The full list of included studies is provided in Table 1 [1, 2, 3, 5, 6, 9, 10, 13, 14, 16, 17, 18, 19, 22, 24, 32, 34, 37, 38, 39, 41, 45].

**Table 1:** Included studies contributing to the primary HRA revision evidence base.

Study	Country/centre	Design	Patients	Hips	Follow‐up	Primary analytical role
Ball 2007	USA	Matched cohort	20	21	Mean 46 months	Femoral‐side failure and cup retention
De Haan 2008	Belgium/Netherlands	Institutional cohort	42	42	Mean 32 months	Malposition, metallosis, dislocation
Grammatopoulos 2009	UK	Retrospective matched cohort	53	53	Mean 3.0 years	ARMD/pseudotumour vs. mechanical failure
de Steiger 2010	Australia	AOANJRR registry	397	397	Registry 5‐year CPR	Early registry benchmark
Gilbert 2010	UK	Institutional cohort	NR	76	Mean 3.8 years	Femoral‐only revision by failure mode
Sandiford 2010	UK	Consecutive cohort	25	25	Mean 12.7 months	Early PROM recovery
Jaiswal 2011	UK	Strategy‐comparative cohort	182	182	3.8–4.5 years by arm	Acetabular‐only vs. femoral‐only vs. both‐component revision
Garrett 2011	UK	Matched cohort	14	14	Mean 49 months	Femoral‐only large‐head conversion
Desloges 2012	Canada	Matched cohort	22	23	Mean 37 months	Revised HRA vs. primary/revision THA
Su & Su 2013	USA	Institutional cohort	NR	55	Mean 2.3 years	Indication‐specific outcomes
Pritchett 2014	USA	Prospective single‐surgeon cohort	89	90	Mean 61 months	One‐component revision for metal wear debris
Wong 2015	Australia	AOANJRR registry	850	884	10‐year registry CPR	Largest registry benchmark
Bouveau 2018	France	Institutional cohort	15	17	Mean 6.7 years	DAA conversion and metal ion reduction
Blevins 2019	USA	Comparative cohort	62	71	Median 1.7 years	Retained cup + dual mobility vs. both‐component revision
Amstutz 2020	USA	Long‐term single‐surgeon cohort	42	43	Mean 8.3 years	Long‐term revised HRA survivorship
Lainiala 2020	Finland	Mixed MoM revision cohort with HRA subgroup	NR	108 HRA subgroup	7‐year survivorship	ARMD‐specific HRA subgroup
Coutandin 2021	Germany	Case series	6	6	Mean 3.25 years	Short‐stem THA after failed HRA
Salmons 2022	USA	Institutional registry cohort	49	52	Mean 6 years	Contemporary revision THA, dislocation, dual mobility
Atallah 2022	Egypt	Prospective case series	35	35	Inconsistently reported	PROM sensitivity evidence
Mutschler 2024	Switzerland/France	Matched registry cohort	17	17	Mean 95 months	Conversion HRA vs. primary/revision THA
Ricard 2024	Canada	Prospective database retrospective review	30	33	Annual follow‐up	HAA revision, reoperations
George 2024	USA	Comparative PROM cohort	36	37	10‐year survivorship	ALTR vs. non‐ALTR PROM signal
Antonioli 2026	USA	Retrospective cohort	40	40	Mean 4.1 years	Retained cup, mostly dual mobility

Abbreviations: ALTR, adverse local tissue reaction; AOANJRR, Australian Orthopaedic Association National Joint Replacement Registry; ARMD, adverse reaction to metal debris; HAA, Hueter‐anterior approach; HRA, hip resurfacing arthroplasty; MoM, metal‐on‐metal; PROM, patient‐reported outcome measure; THA, total hip arthroplasty.

Follow‐up was heterogeneously reported. The weighted mean post‐revision follow‐up across cohort studies was 48.2 months. Follow‐up also differed between revision strategies within individual comparative studies; for example, Jaiswal et al. reported mean follow‐up of 4.5 years after isolated acetabular revision, 3.8 years after isolated femoral revision, and 4.0 years after both‐component revision [22]. In studies where the primary implant was documented, the most frequently revised prosthesis was the BHR (*n* = 304), followed by Conserve Plus (*n* = 41), DePuy ASR (*n* = 25), Cormet (*n* = 7), Durom (*n* = 5), ReCap (*n* = 4) and McMinn (*n* = 2). Full details are presented in Table 2.

**Table 2:** Extractable primary resurfacing device counts.

Primary resurfacing device	Extractable hips	% of specified devices
Birmingham hip resurfacing	304	78.4
Conserve plus	41	10.6
DePuy ASR	25	6.4
Cormet	7	1.8
Durom	5	1.3
ReCap	4	1.0
McMinn	2	0.5
Total specified	388	100.0

*Note*: Device counts are restricted to studies with extractable, non‐overlapping primary implant data. Registry‐only or non‐attributable implant data were not assigned to device‐specific denominators.

### Indications for revision

Across studies with extractable indication data, 1689 indication instances were recorded. The largest indication group was metal‐related pathology, including ARMD, ALVAL, metallosis, pseudotumour or metal wear debris (*n* = 524; 31.0%), followed by loosening or osteolysis (*n* = 454; 26.9%) and fracture (*n* = 337; 20.0%). Pain or unexplained symptoms accounted for 94 indication instances (5.6%), component malposition or impingement for 63 (3.7%), avascular necrosis for 59 (3.5%), infection for 18 (1.1%) and instability or dislocation for 27 (1.6%). Grouped indications are shown in Table 3.

**Table 3:** Grouped indications for revision across extractable indication instances.

Indication group	*n*	% of indication instances
Metal‐related pathology/ARMD/ALVAL/metallosis/pseudotumour	524	31.0
Loosening/osteolysis	454	26.9
Fracture	337	20.0
Pain/unexplained symptoms	94	5.6
Component malposition/impingement	63	3.7
Avascular necrosis	59	3.5
Other mechanical/femoral failure	48	2.8
Instability/dislocation	27	1.6
Infection	18	1.1
Excessive wear/synovitis/implant breakage	14	0.8
Other/implant failure/leg length/sizing/tumour/progression	49	2.9
Unspecified/unknown	2	0.1
Total indication instances	1689	100.0

Abbreviations: ALVAL, aseptic lymphocyte‐dominated vasculitis‐associated lesion; ARMD, adverse reaction to metal debris.

The distribution of indications differed substantially by revision strategy. In the AOANJRR registry analysis, femoral‐component revision was predominantly performed for fracture, whereas both‐component revision was most performed for loosening/lysis and metal‐related pathology [4, 45]. Acetabular‐component revision was uncommon and was concentrated in acetabular loosening/lysis and malposition [22, 41, 45].

### Revision strategies and re‐revision rates

Both‐component revision was the most common strategy (828/1366; 60.6%), followed by femoral‐only revision (486/1366; 35.6%). Isolated acetabular revision was uncommon (52/1366; 3.8%). Results are presented in Table 4.

**Table 4:** Strategy‐by‐indication matrix across studies reporting both variables.

Indication	Both‐component revision, *n* (%)	Femoral‐only/cup retained, *n* (%)	Retained cup + dual mobility, *n* (%)	Acetabular‐only/one‐component, *n* (%)	Total
Loosening/lysis/osteolysis	268 (64.4)	90 (21.6)	24 (5.8)	34 (8.2)	416
ARMD/ALVAL/metallosis/pseudotumour/metal‐related	245 (66.6)	2 (0.5)	44 (12.0)	77 (20.9)	368
Fracture	76 (23.4)	239 (73.5)	10 (3.1)	0 (0.0)	325
Pain/unexplained symptoms	46 (69.7)	17 (25.8)	2 (3.0)	1 (1.5)	66
Osteonecrosis/AVN	16 (27.1)	43 (72.9)	0 (0.0)	0 (0.0)	59
Other/implant failure/mixed non‐specific	39 (72.2)	11 (20.4)	1 (1.9)	3 (5.6)	54
Instability/dislocation	18 (66.7)	6 (22.2)	0 (0.0)	3 (11.1)	27
Malposition/impingement	9 (39.1)	4 (17.4)	0 (0.0)	10 (43.5)	23
Infection	17 (100.0)	0 (0.0)	0 (0.0)	0 (0.0)	17
Wear/synovitis/acetabular breakage	10 (90.9)	1 (9.1)	0 (0.0)	0 (0.0)	11
Implant or head breakage	1 (25.0)	3 (75.0)	0 (0.0)	0 (0.0)	4
Unspecified	2 (100.0)	0 (0.0)	0 (0.0)	0 (0.0)	2
Total	747 (54.4)	416 (30.3)	81 (5.9)	128 (9.3)	1372

Abbreviations: ALVAL, aseptic lymphocyte‐dominated vasculitis‐associated lesion; ARMD, adverse reaction to metal debris.

Across 1705 revised hips with extractable longitudinal outcome data, 157 re‐revisions or major reoperations were recorded, giving an overall event rate of 9.2%. In the strategy‐specific data set, both‐component revision had the lowest re‐revision rate at 7.1% (59/828). Femoral‐only revision had an intermediate re‐revision rate of 13.0% (63/486), while isolated acetabular revision had the highest rate at 19.2% (10/52).

The higher event proportion after femoral‐only revision must also be interpreted in the context of indication mix. In the strategy‐by‐indication matrix, femoral‐only or cup‐retained revision was used in 239/325 fracture revisions (73.5%), whereas both‐component revision predominated in metal‐related pathology (245/368; 66.6%) and loosening/lysis/osteolysis (268/416; 64.4%). In the AOANJRR registry, fracture accounted for 191/310 femoral‐component revisions, while metal‐related pathology and loosening/lysis accounted for 186/537 and 215/537 combined‐component revisions, respectively [4, 45]. Thus, strategy‐specific re‐revision rates reflect both surgical construct and the indication for which that construct was selected [22, 45].

Time‐to‐event analyses, though, did not reproduce a simple hierarchy based on these crude event rates. De Steiger et al. reported 5‐year cumulative re‐revision of 20% after acetabular‐only revision, 7% after femoral‐only revision, and 5% after both‐component revision [41]. Jaiswal et al. reported 10‐year survivorship of 75%, 93% and 96%, respectively [22]. In the later AOANJRR analysis of Wong et al., however, the overall 10‐year cumulative re‐revision was 26% and adjusted comparisons showed no significant difference in re‐revision hazard between acetabular‐only, femoral‐only and both‐component revision [45].

At study level, re‐revision and major reoperation were reported separately wherever the source permitted. Wong et al. reported 102 further component revisions among 884 revised HRAs, with crude re‐revision rates of 18.9% after acetabular‐component revision, 16.5% after femoral‐component revision and 8.2% after combined‐component revision [45]. Salmons et al. reported 5‐year survivorship free from re‐revision of 89% after revision THA for failed MoM HRA, with six re‐revisions and dislocation as the dominant failure mechanism [38]. Ricard et al. reported five major reoperations among 33 anterior‐approach revisions, including three infections, one acetabular loosening, and one trunnionosis [37]. Blevins et al. reported six additional revisions among 71 conversions, including two retained‐cup dual‐mobility failures due to intraprosthetic dislocation and polyethylene wear [6].

### Bearing construct and acetabular component retention

Bearing choice was closely linked to component strategy. Earlier femoral‐only revisions often used large metal heads against retained metal resurfacing cups [5, 16, 28]. More recent retained‐cup strategies have increasingly used dual‐mobility constructs to eliminate the MoM articulation while preserving the acetabular shell and improving stability [2, 6, 15, 31]. Bearing construct and acetabular component management are summarised in Table 5.

**Table 5:** Bearing construct and acetabular component management.

Revision setting	Construct	Key studies	Reported denominator/outcome	Key interpretation
Femoral‐only revision with retained MoM cup	Large metal head matched to retained metal resurfacing shell	Ball, Garrett, selected older series	Strategy included within femoral‐only/cup‐retained group	Preserves acetabular bone stock but maintains or recreates a large‐head metal articulation
Retained cup + dual mobility	Dual‐mobility articulation within retained monoblock HRA shell	Blevins, Antonioli	67 hips across contemporary retained‐cup DM series; 8/67 events (11.9%)	Eliminates MoM articulation and improves effective head size, but carries IPD, polyethylene wear, infection and femoral‐sided failure risk
Retained cup + dual mobility, comparative cohort	RAC + DM vs. both‐component revision	Blevins	27 RAC + DM vs. 44 both‐component; 2/27 RAC + DM failures through IPD/polyethylene wear	Short‐term function and metal ion reduction comparable, but retained‐cup failures were construct‐specific
Retained cup, mostly dual mobility	Retained shell with predominantly DM articulation	Antonioli	40 retained‐cup conversions; 39/40 DM; acetabular survivorship 100%; 6 reoperations/revisions	Acetabular component durable, but reoperations included three DAIRs, one IPD head/liner exchange, and two femoral stem revisions
Both‐component revision	Stemmed THA with revised acetabular component and contemporary bearing	Wong, Jaiswal, Blevins, Salmons, Ricard	59/828 events in strategy‐specific data set (7.1%)	Removes MoM articulation and addresses cup pathology
Isolated acetabular revision	Revised acetabular component with retained femoral resurfacing component	Jaiswal, Wong	10/52 events (19.2%)	Highest observed rate; small denominator
Selected one‐component revision for metal wear debris	Component‐preserving revision, acetabular‐sided or femoral‐sided	Pritchett	3/90 failures (3.3%); no dislocations	Specialist selected cohort; not equivalent to historical isolated acetabular revision
Repeat resurfacing	Second HRA	Jaiswal/Amstutz selected cases	Rare	Limited contemporary evidence

Abbreviations: HRA, hip resurfacing arthroplasty; MoM, metal‐on‐metal.

Retained‐cup reconstruction was reported primarily in patients in whom the acetabular component was well fixed, well positioned, undamaged, and compatible with the planned articulation [2, 6, 18]. Blevins et al. reported 27 retained acetabular component revisions, compared with 44 both‐component revisions; dual mobility was used in all retained‐cup cases and in 9/44 both‐component revisions. Retained acetabular component dual‐mobility reconstruction and both‐component revision produced comparable short‐term functional improvement and metal ion reduction, but 2/27 retained‐cup cases failed through intraprosthetic dislocation with extensive polyethylene wear, requiring acetabular revision [6]. Antonioli et al. reported 40 retained‐cup conversions, 39/40 using dual mobility. Acetabular component survivorship remained 100% through 4.1 years of follow‐up, but six reoperations or revisions occurred: three infection‐related DAIR procedures, one head‐and‐liner exchange for intraprosthetic dislocation and two femoral stem revisions for aseptic loosening [2].

### Serum metal ion normalisation

Two studies reported paired pre‐ and post‐revision cobalt and chromium concentrations (Table 6). In Bouveau et al., mean cobalt decreased from 35.8 to 4.7 µg/L and chromium from 33.2 to 5.8 µg/L [9]. Blevins et al. reported median chromium decreasing from 5.4 to 1.6 ppb and cobalt from 5.4 to 3.7 ppb in the analysed cohort, with significant reductions reported in both retained‐cup and both‐component revision groups. This study also found no significant difference in post‐revision cobalt or chromium levels between retained acetabular component dual‐mobility reconstruction and both‐component revision [14]. Both studies therefore demonstrated a postoperative reduction in metal ions, but were not combined due to differing underlying summary measures and patient populations.

**Table 6:** Serum metal ion changes after revision.

Analysis	Studies included	*n* hips	Pre‐revision value	Post‐revision value	Absolute reduction	Relative reduction
Cobalt	Bouveau	17	35.8 µg/L	4.7 µg/L	−31.1 µg/L	−86.9%
Chromium	Bouveau	17	33.2 µg/L	5.8 µg/L	−27.4 µg/L	−82.5%
Cobalt	Blevins	68	5.4 ppb	3.7 ppb	−1.7 ppb	−31.5%
Chromium	Blevins	68	5.4 ppb	1.6 ppb	−3.8 ppb	−70.4%

*Note*: Bouveau reported means, whereas Blevins reported medians after exclusion of patients with contralateral HRA. Estimates are therefore presented separately and were not statistically pooled.

Abbreviation: HRA, hip resurfacing arthroplasty.

### PROMs

PROMs improved after revision in studies with extractable paired data, although reporting was heterogeneous across Harris Hip Score (HHS), Oxford Hip Score (OHS), Western Ontario and McMaster Universities Osteoarthritis Index (WOMAC), University of California, Los Angeles (UCLA) activity score, Postel‐Merle d'Aubigné score, Hip disability and Osteoarthritis Outcome Score (HOOS), SF‐12, mHHS and Merle d'Aubigné scores [3, 22, 34, 39]. In the sample‐size‐weighted descriptive analysis, weighted mean HHS improved from 64.4 to 92.5 points [34, 39]. Strategy‐specific PROM comparisons were limited and confounded by indication; detailed PROM synthesis is provided in the Supporting Information [17, 19, 22, 34, 42].

### Complications and reoperations

Across the studies, 118 reportable events were reported (Table 7). Instability, dislocation or intraprosthetic dislocation was the most common category (*n* = 32; 27.1%), followed by infection or periprosthetic joint infection (PJI) (*n* = 24; 20.3%), neurovascular or systemic complications (*n* = 14; 11.9%), loosening or mechanical fixation failure (*n* = 13; 11.0%) and fracture (*n* = 12; 10.2%). Metal‐related recurrence, residual pseudotumour or ion‐related events accounted for six events (5.1%).

**Table 7:** Reported complication‐event categories.

Complication category	*n* events	% of reported events
Instability/dislocation/intraprosthetic dislocation	32	27.1
Infection/PJI/DAIR	24	20.3
Neurovascular/systemic complications	14	11.9
Loosening/mechanical fixation failure	13	11.0
Fracture	12	10.2
Other/unspecified major complication	9	7.6
Soft‐tissue/other local complication	6	5.1
Metal‐related recurrence/residual pseudotumour/ion‐related event	6	5.1
Pain/other	1	0.8
Trunnionosis/corrosion/implant wear	1	0.8
Total reported events	118	100.0

Abbreviations: DAIR, debridement, antibiotics and implant retention; PJI, periprosthetic joint infection.

Registry data provided the most robust population‐level map of re‐revision mechanisms (Table 8). In Wong et al., 102 further revisions were attributed to loosening/lysis (*n* = 29), metal‐related pathology (*n* = 22), infection (*n* = 19), dislocation (*n* = 19), fracture (*n* = 5), malposition (*n* = 3), leg length discrepancy (*n* = 2), pain (*n* = 2) and other causes (*n* = 1) [45].

**Table 8:** Study‐level complications and reoperations.

Study	*n* hips	Revision context	Subsequent surgery	Infection/PJI	Instability/dislocation/IPD	Loosening/mechanical failure	Other complications	Key interpretation
Grammatopoulos	53	Pseudotumour, fracture, other failure	10 confirmed further revisions + 1 awaiting	3 infections in fracture group	3 recurrent dislocations	2 loosenings	3 femoral nerve palsies; 1 femoral artery stenosis; 50% major complications in pseudotumour group	ARMD/pseudotumour behaves as high‐risk biological failure
De Haan	42	Malposition/metallosis revision	4 further revisions	NR	4 dislocations	Loosening/protrusion among second revisions	NR	Malposition/metallosis carries instability/revision risk
Ball	21	Failed femoral component	0 aseptic loosening/dislocation	0	0	0	1 intraoperative femur fracture; transient femoral nerve palsy; myocardial infarction	Selected femoral‐only conversion can be safe
Gilbert	76	Isolated femoral revision	4 complications/revisions	3 deep infections	0 reported	1 uncemented stem loosening	NR	Femoral‐only revision is not complication‐free
Sandiford	25	Conversion to THA	0 implant failures reported	0 new infections	0 reported	0 reported	2 transient stiffness	Early recovery favourable in short follow‐up
Jaiswal	182	Acetabular‐only, femoral‐only, both‐component	9 re‐revisions	4 infection‐related re‐revisions	3 dislocation‐related re‐revisions	1 femoral loosening	1 fracture	Acetabular‐only had less favourable survival signal
Pritchett	90	One‐component revision for metal wear debris	3 failures requiring re‐revision	1 deep infection	0 dislocations	1 failure/loosening signal	Continuing metallosis in one case	Selected one‐component revision can be successful
Wong	884	AOANJRR registry benchmark	102 further revisions	19 infection re‐revisions	19 dislocation re‐revisions	29 loosening/lysis re‐revisions	22 metal‐related; 5 fracture; 3 malposition; 2 LLD; 2 pain; 1 other	Population‐level re‐revision mechanism map
Bouveau	17	DAA conversion	0 further surgery	0/NR	0/NR	0 further surgery	1 greater trochanter fracture; 4 LFCN dysesthesias	Approach‐specific nerve symptom profile
Blevins	71	RAC + DM vs. both‐component	6 additional revisions	BC PJI signal	2 RAC IPD/polyethylene‐wear failures; 1 closed reduction	BC osseointegration failure	Acetabular fracture in BC group	Cup retention avoids acetabular morbidity but can fail via IPD/poly wear
Amstutz	43	Long‐term mixed revised HRA	6 re‐revisions	1 late PJI/DAIR	3 dislocations	2 acetabular loosenings plus other mechanical failures	Nerve palsy, wound issue, MI	Long‐term failure includes late metal/corrosion and mechanical issues
Lainiala	108	HRA subgroup revised for ARMD	9 re‐revisions	1 infection re‐revision	Dislocation re‐revisions and closed reductions	1 stem loosening	Residual/recurrent ARMD, DVT, fascial rupture	ARMD subgroup durable but early instability/fracture/infection dominate
Salmons	52	Contemporary revision THA	6 re‐revisions; 8 reoperations	1 acute PJI/DAIR	9 dislocations total; 4 revised	Retained cup debonding/loosening signal	PE; iliopsoas impingement reoperation	Instability is dominant failure mode
Antonioli	40	Retained cup, mostly DM	6 reoperations/revisions	3 DAIRs for infection	1 head/liner exchange for IPD	2 femoral stem revisions	ED visits/readmissions	Retained cup durable, but PJI/reoperation burden relevant

Abbreviations: ARMD, adverse reactions to metal debris; DAIR, debridement, antibiotics and implant retention; HRA, hip resurfacing arthroplasty; PJI, periprosthetic joint infection; THA, total hip arthroplasty.

Biological failure carried a distinct complication profile. Grammatopoulos et al. reported a 50% major complication rate after pseudotumour revision, including recurrent dislocation, femoral nerve palsy, femoral artery stenosis, loosening, recurrent pseudotumour and further revision. In contrast, fracture and other non‐pseudotumour revisions had better functional outcomes and lower major complication rates [19].

Instability was a recurring failure mode in contemporary conversion series. Salmons et al. reported that dislocation accounted for four of six re‐revisions, with a 5‐year cumulative dislocation probability of 19% [38]. Retained‐cup dual‐mobility reconstruction was associated with fewer conventional dislocations in available selected cohorts but introduced construct‐specific risks: Blevins et al. reported intraprosthetic dislocation with polyethylene wear in two retained‐cup cases, while Antonioli et al. reported one head‐and‐liner exchange for intraprosthetic dislocation [2, 6]. Thus, dual mobility should be analysed as a distinct retained‐cup construct rather than grouped generically under femoral‐only conversion [2, 6, 38].

### CoC resurfacing

Lin et al. reported the 2‐year clinical and radiographic results of the first 200 consecutive ReCerf CoC HRAs in a prospective multicentre cohort (mean follow‐up 42.5 months; 185 completing 2‐year PROM follow‐up). Significant improvements were demonstrated in all PROMs, including OHS, HOOS Jr., WOMAC, Forgotten Joint Score and UCLA activity score (all *p* < 0.001). No revisions were reported at 2 years in the primary cohort [27].

Koh et al. reported 5‐year survivorship of the ReCerf in an international retrospective series of 604 hips (336 males, 268 females; mean age 50.1 years). Kaplan–Meier survivorship at 5 years was 98.0% (95% confidence interval: 96.3–99.0); 128 hips with 5‐year follow‐up [23]. Reported failures in primary CoC resurfacing series to date appear early and predominantly mechanical, particularly peri‐prosthetic femoral neck fracture, but revision strategies and outcomes after failed CoC HRA have not yet been reported [23, 27].

## DISCUSSION

This systematic review of 23 studies and 2320 reported HRA revision procedures shows that revision outcomes vary according to indication and reconstruction strategy. The dominant complications were instability and infection. Clinical decision‐making therefore centres on whether one component can reasonably be retained or whether both components require revision to address the mechanical, tribological or biological driver of failure. The available evidence supports structured assessment of whether a component can reasonably be retained, but does not provide comparative evidence sufficient to prescribe a universally superior revision strategy. Figure 2 therefore presents a decision framework derived from the reported indications and construct‐specific considerations.

**Figure 2:**
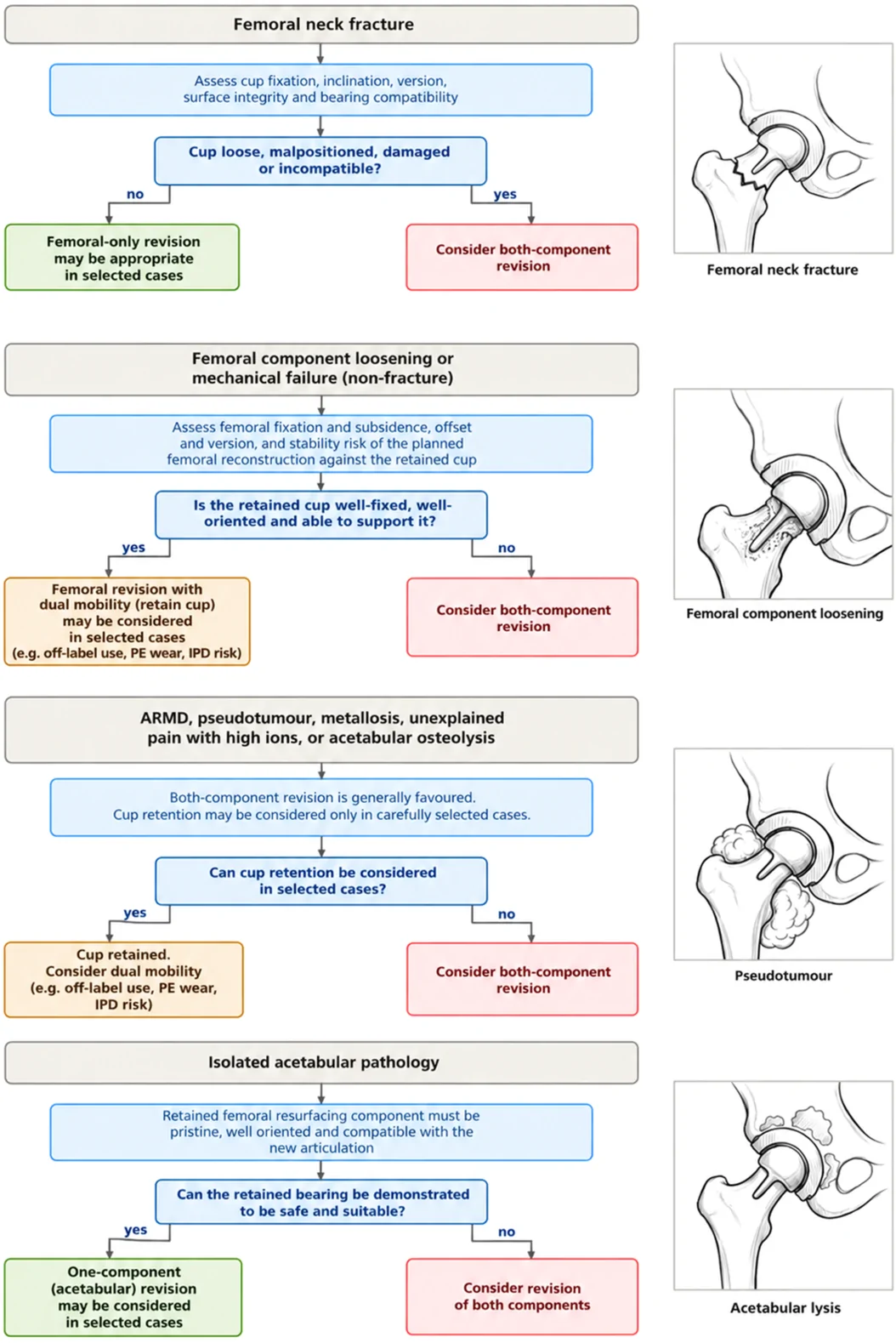
Proposed decision algorithm for revision of failed hip resurfacing arthroplasty. ARMD, adverse reaction to metal debris.

### Revision strategies

Both‐component revision had the most favourable crude strategy‐specific outcome, with 59 re‐revisions or major reoperations among 828 hips (7.1%), compared with 63/486 (13.0%) after femoral‐only or cup‐retained revision and 10/52 (19.2%) after isolated acetabular revision [2, 6, 22, 45]. However, these figures should not be read as a simple hierarchy of surgical quality, because follow‐up differed between and within studies, component strategy was strongly indication‐dependent, and the underlying reports variably used re‐revision and major reoperation as endpoints. Femoral‐only or cup‐retained revision was used in 239/325 fracture revisions (73.5%), whereas both‐component revision predominated for metal‐related pathology (245/368; 66.6%) and loosening/lysis/osteolysis (268/416; 64.4%) [4, 22, 45]. This distinction is particularly important because time‐to‐event analyses were not entirely concordant with the crude proportions. Strategy should therefore be interpreted in conjunction with indication, component status, and time at risk rather than through the crude event proportions alone.

The practical clinical consideration suggested by the observational literature is that in isolated femoral fracture or femoral loosening, cup retention may be appropriate [5, 16, 18]. In ARMD, pseudotumour, acetabular malposition, osteolysis, visible bearing damage or unexplained metal‐related symptoms, the retained acetabular component must additionally be assessed for involvement in the mechanical or biological failure process [13, 19, 22, 45].

When the acetabular component is loose, malpositioned, damaged, infected or implicated in metal‐related pathology, both‐component revision permits removal of the femoral resurfacing component and acetabular shell, elimination of the MoM articulation, correction of cup orientation, treatment of acetabular loosening or osteolysis and use of a contemporary bearing [13, 22, 45]. Both‐component revision was consequently used frequently for metal‐related pathology and loosening/lysis in the included literature. However, its lower crude event rate poses questions about whether or not the strategy itself was the root cause for the lower risk of subsequent surgery.

This is particularly important in ARMD. A retained cup may appear well fixed yet still be malpositioned, scratched, edge‐loaded, worn or biologically unsafe [13, 19]. Fixation alone is therefore insufficient to determine suitability for retention. In ARMD, the goal is not simply to insert a femoral stem; it is to remove the metal‐debris source, reconstruct a stable articulation, and prevent recurrent biological insult in a hip that may already have abductor and capsular compromise [13, 19].

The AOANJRR data support this surgical logic. Wong et al. reported 102 further revisions among 884 revised HRAs, with crude re‐revision rates of 8.2% after combined‐component revision, compared with 16.5% after femoral‐component revision and 18.9% after acetabular‐component revision [45]. This does not mean both‐component revision is mandatory for every failed HRA, but it does support a lower threshold for cup removal if there is any suspicion that the acetabular‐component could be loose, malpositioned, infected, damaged, or involved in metal‐debris pathology [13, 45]. Conversely, when failure is purely femoral, and the acetabular shell is well fixed, well oriented, undamaged and biologically uninvolved, cup removal may add bone loss and morbidity without proportional benefit [2, 5, 6, 15, 16, 18].

Isolated acetabular revision was uncommon and had a crude event rate of 19.2%, but the denominator was small, and the available evidence does not support treating this proportion as an intrinsic treatment‐specific risk [22, 45]. Interpretation also depends on the mechanism of failure. Revising the cup while retaining a previously loaded femoral resurfacing component creates a new articulation around an old bearing surface. That femoral component may have undergone edge‐loading, scratching, metal transfer, corrosion, or subclinical deformation causing altered clearance [13, 25, 43]. Notably, Pritchett reported 90 one‐component revisions for ARMD, with just three failures requiring re‐revision, one deep infection and no dislocations at a mean of 61 months [34]. Patients with a smaller head size received a unique two‐component titanium shell polyethylene liner component, while the remainder received a like‐for‐like metal resurfacing cup or a dual‐mobility construct. This series demonstrates that selected one‐component reconstruction can produce favourable results and further cautions against inferring outcome solely from revision category [34].

Femoral‐only revision (by converting the resurfacing to a stemmed implant) is commonly indicated for femoral neck fracture, isolated femoral loosening or femoral head collapse with a stable and well‐positioned cup; conversion can be closer to a primary THA than to a complex revision THA [5, 16, 18, 39].

However, in the setting of ARMD, the abductors may be necrotic, the capsule attenuated, the acetabulum osteolytic, and the retained cup part of the metal‐debris mechanism [1, 19]. These biological differences confound comparisons between femoral‐only revision and strategies used for other failure modes [22, 45]. Second, earlier femoral‐only revisions often used large cobalt‐chromium heads against retained MoM resurfacing cups, before the widespread adoption of dual‐mobility constructs [5, 16, 28]. If the retained shell is malpositioned, edge‐loaded, scratched or implicated in ARMD, placing a large metal head into that shell may perpetuate metal wear rather than resolve it [13, 25, 28, 43]. Further, a large metal head on a stem also produces frictional torque at the modular head‐neck junction, raising concern for mechanically assisted crevice corrosion and trunnionosis [28].

As such, contemporary reports increasingly describe dual‐mobility constructs in place of large‐head metal articulations for femoral‐only revisions [2, 6, 15, 20, 31, 40, 44]. This eliminates the MoM articulation and increases effective head size in hips at risk of instability. This is especially attractive in ARMD, where abductors and capsule may be compromised, and in patients where removing a well‐fixed shell would cause major acetabular bone loss [2, 6, 15, 20, 31, 40, 44].

The trade‐off is that a metal resurfacing shell was not designed as a polyethylene‐bearing dual‐mobility socket, raising concerns that the rim geometry, clearance, surface finish and wear environment are incompatible [2, 6]. The clinical data reflect this compromise: Blevins et al. compared 27 retained‐cup dual‐mobility revisions with 44 both‐component revisions. Function and metal ions improved in both groups, but 2/27 (7.4%) retained‐cup dual‐mobility cases failed through intraprosthetic dislocation with extensive polyethylene wear, requiring acetabular revision [6]. Antonioli et al. reported more reassuring results on 40 retained‐cup conversions, 39/40 using dual mobility: there was 100% acetabular component survivorship, but six reoperations or revisions, including three DAIRs, one head‐and‐liner exchange for intraprosthetic dislocation and two femoral stem revisions [2].

These data support retained‐cup dual mobility as a component‐preserving option in selected patients rather than demonstrating superiority over full component revision. The available series predominantly used it when the cup was well fixed, acceptably oriented and undamaged, particularly where acetabular removal would have been undesirable, or instability risk was high [2, 6].

### ARMD revision

Metal‐related pathology (ARMD/ALVAL/metallosis/pseudotumour) was the most common indication for HRA revision, accounting for 524/1689 indication instances (31.0%) [13, 19, 22, 45]. Patients revised for pseudotumour had substantially worse function than those revised for fracture or other causes, with OHS 20.9 after pseudotumour revision compared with 40.2 after fracture and 37.8 after other non‐pseudotumour failure [19].

Revision substantially reduced systemic metal burden. Both studies providing paired metal‐ion data demonstrated postoperative reductions [6, 9]. In Bouveau et al., cobalt decreased from 35.8 to 4.7 µg/L and chromium from 33.2 to 5.8 µg/L [9]. Blevins et al. found significant reductions in both retained‐cup dual‐mobility and both‐component revision groups, with no significant difference in post‐revision cobalt or chromium [6]. These findings suggest that elimination of the MoM articulation can reduce systemic metal exposure even in selected retained‐cup reconstructions, but they do not demonstrate equivalence between revision strategies [6, 9]. Normal ions do not prove that the retained cup is mechanically safe; Grammatopoulos et al. reported a 50% major complication rate after HRA pseudotumour revision, which may reflect the association of pseudotumours with abductor damage, instability, nerve injury, vascular compromise and recurrent soft‐tissue disease [1, 19].

Patient counselling should reflect this difference. Femoral neck fracture after HRA can often be framed as a relatively straightforward conversion with preserved femoral bone stock [5, 16, 39]. ARMD with abductor damage should be counselled more like complex revision THA, with instability, infection, persistent weakness and incomplete recovery as central risks [1, 19, 38].

### CoC resurfacing

The revision evidence in this review is overwhelmingly MoM‐based. Non‐MoM resurfacing may change the revision landscape by removing ARMD, metal ion surveillance and cobalt/chromium biology [23, 27, 29]. Lin et al. reported 200 ReCerf CoC HRAs, with significant PROM improvement and no revisions at 2 years, while Koh et al. reported 98.0% 5‐year survivorship in 604 ReCerf hips, with no significant effect of sex or femoral head diameter on revision risk [23, 27]. These are primary survivorship data and must not be interpreted as evidence regarding outcomes after revision of failed CoC resurfacing. Reported early failures appear predominantly mechanical, but whether this will translate into different revision indications or outcomes remains unknown. Revision strategies and re‐revision outcomes after failed CoC HRA have not yet been reported. Future CoC studies should therefore report not only primary survivorship but failure indication, component retention, bearing choice at revision, complications and subsequent re‐revision [23, 27, 29].

### Limitations

This review is limited by its observational evidence base, absence of randomised comparisons, and confounding by indication, component fixation, component position, soft‐tissue status, surgeon experience and patient demand. Revision strategy was therefore not randomly or comparably allocated, and differences in crude strategy‐specific event proportions cannot be interpreted as causal treatment effects. PROMs, follow‐up, complications and survivorship were reported heterogeneously. Follow‐up was particularly important for re‐revision: simple event proportions combine cohorts observed for substantially different durations and cannot substitute for time‐to‐event analysis. Strategy‐specific follow‐up was unavailable in many studies, preventing valid time‐standardisation across the complete data set. Definitions of re‐revision and major reoperation also varied. These outcomes were separated at study level where possible; where the source did not permit separation, events were retained only as a composite re‐revision‐or‐major‐reoperation endpoint. Registry and institutional cohorts may overlap, so registry data should be interpreted as population‐level context rather than additive denominators. Metal‐ion studies used incompatible summary statistics and were therefore presented individually rather than pooled. Finally, although CoC resurfacing is discussed contextually, revision outcomes after failed non‐MoM resurfacing remain largely unreported.

## CONCLUSION

Revision of failed HRA can provide meaningful functional recovery and substantial metal ion reduction, but outcomes depend on failure mechanism, retained component status and bearing choice. When the acetabular component is loose, malpositioned, damaged, infected or implicated in metal‐related failure, both‐component revision permits the implicated acetabular component and MoM articulation to be removed. Conversely, component retention may be considered when failure is isolated to the opposite side, and the retained component is fixed, acceptably oriented, undamaged and biologically uninvolved. Retained‐cup dual mobility is a component‐preserving option in selected patients, trading acetabular preservation and stability against polyethylene wear, intraprosthetic dislocation, infection and uncertain long‐term construct behaviour. Evidence for isolated acetabular revision remains limited and derives from small, highly selected cohorts. Revision strategies for the new generation of non‐MoM resurfacing devices will be guided by meticulous data collection on indication, component retention, bearing construct, soft‐tissue status, complications and re‐revision at patient level.

## AUTHOR CONTRIBUTIONS

**Srikar Reddy Namireddy**: Conceptualisation; methodology; data curation; formal analysis; investigation; writing—original draft; writing—review and editing. **Hariharan Subbiah Ponniah**: Data curation; investigation; writing—review and editing. **Milos Brjklac**: Data curation; investigation; writing—review and editing. **Justin Cobb**: Supervision; writing—review and editing. **Kartik Logishetty**: Conceptualisation; methodology; supervision; writing—review and editing.

## FUNDING INFORMATION

The authors have no funding to report.

## CONFLICT OF INTEREST STATEMENT

Kartik Logishetty reports grants from Orthopaedic Research UK and the Royal College of Surgeons of England; consulting fees from JRI and Johnson & Johnson; speaker payments or honoraria from JRI and Smith & Nephew; support for attending meetings and travel from JRI; and a leadership or fiduciary role and stock or stock options in Embed Biotech. These disclosures are unrelated to the present study. Justin Cobb reports institutional grants from JRI, DePuy, Smith & Nephew, Zimmer Biomet and the Sir Michael Uren Foundation; royalties or licences from MatOrtho and Embody Orthopaedic; consulting fees from JRI, DePuy and Zimmer Biomet; speakers bureau payments from Zimmer Biomet and CeramTec; and stock or stock options in Embody Orthopaedic, Orthonika and Additive Instruments. Justin Cobb is also Director of Embody Orthopaedic. These disclosures are unrelated to the present study. The other authors declare no conflicts of interest.

## ETHICS STATEMENT

The authors have nothing to report.

## Supporting information

Supporting File 1.

## Data Availability

The data that support the findings of this study are available from the corresponding author upon reasonable request.
