# Supplementary material for: Revision outcomes after failed hip resurfacing arthroplasty vary by failure indication and component strategy: A systematic review
Source: J Exp Orthop. 2026 Nov 3;13(4):e70965. doi: 10.1002/jeo2.70965 (PMC13729969; doi:10.1002/jeo2.70965)
Supplement: Supplementary file 1 [file JEO2-13-e70965-s001.docx]

# **Supplementary Materials**

In the HHS analysis, including in studies by Sandiford and Pritchett, weighted mean HHS improved from 64.4 to 92.5 points, a mean improvement of 28.0 points[28,29]. Including Atallah’s study as sensitivity evidence yielded a similar postoperative HHS of 92.4, with a larger improvement of 35.1 points because baseline function was lower[35]. WOMAC improved from 67.4 to 9.0 in studies with extractable paired data, while harmonised OHS improved from 20.0 to 41.0 after adjusting Sandiford’s older OHS scoring direction to the modern higher-is-better scale. UCLA activity improved from 3.6 to 8.1.[29,35]. Findings are summarised in Supplementary Table 1.

Strategy-specific PROM comparisons were limited. Jaiswal et al. reported median HHS values of 74 after isolated acetabular revision, 82 after isolated femoral revision, and 85 after both-component revision, with no statistically significant difference between groups[11]. However, these groups differed by failure indication, limiting causal interpretation. Indication-specific studies suggested that failure biology mattered substantially: Grammatopoulos et al. reported markedly worse OHS after pseudotumour revision than after fracture or other non-pseudotumour failure, while Su & Su and George et al. similarly reported poorer outcomes after unexplained pain, metal sensitivity, or ALTR than after mechanical failure[24,27,38].

***Supplementary Table 1.*** *Restricted paired PROM analysis*

| PROM | Studies included | n hips | Pre-revision weighted mean | Post-revision weighted mean | Change | Interpretation |
| --- | --- | --- | --- | --- | --- | --- |
| HHS, conservative analysis | Sandiford, Pritchett | 115 | 64.4 | 92.5 | +28.0 | Substantial functional improvement after revision |
| HHS, sensitivity analysis | Sandiford, Pritchett, Atallah | 150 | 57.3 | 92.4 | +35.1 | Similar postoperative HHS; larger change due to lower baseline in Atallah |
| WOMAC | Sandiford, Atallah | 60 | 67.4 | 9.0 | −58.4 | Marked symptom/function improvement; lower score indicates better outcome |
| OHS, harmonised direction | Sandiford transformed + Atallah | 60 | 20.0 | 41.0 | +20.9 | Marked improvement after harmonising scoring direction |
| UCLA activity | Sandiford, Atallah | 60 | 3.6 | 8.1 | +4.4 | Activity improved substantially |
| PMA | Bouveau only | 17 | 9 | 16 | +7 | Approach-specific supportive evidence only |

# **SUPPLEMENTARY REFERENCES**

References cited in the Supplementary Materials correspond to the numbering used in the main manuscript reference list.

11.	Jaiswal A, Gilbert RE, Kumar KHS, Carrothers AD, Kuiper JH, Richardson JB. Function and survival after revision of hip resurfacing. Hip Int. 2011;21(5):610–615.
24.	Grammatopoulos G, Pandit H, Kwon YM, et al. Hip resurfacings revised for inflammatory pseudotumour have a poor outcome. J Bone Joint Surg Br. 2009;91-B(8):1019–1024.
27.	Su EP, Su SL. Surface replacement conversion: results depend upon reason for revision. Bone Joint J. 2013;95-B(11 Suppl A):88–91.
28.	Pritchett JW. One-component revision of failed hip resurfacing from adverse reaction to metal wear debris. J Arthroplasty. 2014;29(1):219–224.
29.	Sandiford NA, Muirhead-Allwood SK, Skinner JA. Revision of failed hip resurfacing to total hip arthroplasty rapidly relieves pain and improves function in the early postoperative period. J Orthop Surg Res. 2010;5:88.
30.	Bouveau V, Haen TX, Poupon J, Nich C. Outcomes after revision of metal-on-metal hip resurfacing to total arthroplasty using the direct anterior approach. Int Orthop. 2018;42(11):2543–2549.
35.	Atallah AHA, Hassan Wahd YES, Abdel-AAl MA. Hip resurfacing arthroplasty failure. Conversion to total hip: is it a favorable solution with satisfactory outcomes? J Orthop Sports Med. 2022;4(1):162–177.
38.	George J, Taylor AJ, Schmalzried TP. Examining the revisability benefit of hip resurfacing arthroplasty. World J Orthop. 2024;15(6):554–559.
